# The Dynamic Processing of CD46 Intracellular Domains Provides a Molecular Rheostat for T Cell Activation

**DOI:** 10.1371/journal.pone.0016287

**Published:** 2011-01-19

**Authors:** Siobhan Ni Choileain, Nathan J. Weyand, Christian Neumann, Joelle Thomas, Magdalene So, Anne L. Astier

**Affiliations:** 1 Institute of Immunology and Infection Research, Edinburgh, United Kingdom; 2 Centre for Inflammation Research, Centre for Multiple Sclerosis Research, Queen's Medical Research Institute, University of Edinburgh, Edinburgh, United Kingdom; 3 BIO5 Institute and Department of Immunobiology, University of Arizona, Tucson, Arizona, United States of America; 4 Université Lyon 1, Lyon, CNRS, UMR5534, Centre de Génétique Moléculaire et Cellulaire, Villeurbanne, France; University Paris Sud, France

## Abstract

**Background:**

Adequate termination of an immune response is as important as the induction of an appropriate response. CD46, a regulator of complement activity, promotes T cell activation and differentiation towards a regulatory Tr1 phenotype. This Tr1 differentiation pathway is defective in patients with MS, asthma and rheumatoid arthritis, underlying its importance in controlling T cell function and the need to understand its regulatory mechanisms. CD46 has two cytoplasmic tails, Cyt1 and Cyt2, derived from alternative splicing, which are co-expressed in all nucleated human cells. The regulation of their expression and precise functions in regulating human T cell activation has not been fully elucidated.

**Methodology/Principal Findings:**

Here, we first report the novel role of CD46 in terminating T cell activation. Second, we demonstrate that its functions as an activator and inhibitor of T cell responses are mediated through the temporal processing of its cytoplasmic tails. Cyt1 processing is required to turn T cell activation on, while processing of Cyt2 switches T cell activation off, as demonstrated by proliferation, CD25 expression and cytokine secretion. Both tails require processing by Presenilin/γSecretase (P/γS) to exert these functions. This was confirmed by expressing wild-type Cyt1 and Cyt2 tails and uncleavable mutant tails in primary T cells. The role of CD46 tails was also demonstrated with T cells expressing CD19 ectodomain-CD46 C-Terminal Fragment (CTF) fusions, which allowed specific triggering of each tail individually.

**Conclusions/Significance:**

We conclude that CD46 acts as a molecular rheostat to control human T cell activation through the regulation of processing of its cytoplasmic tails.

## Introduction

Proper functioning of the immune system depends not only on a rapid, effective activation of immune cells, but also on timely downregulation of the response. Inadequate termination of these responses could lead to autoimmunity, chronic inflammation and cancer. Though the parameters of T cell activation are well documented, mechanisms that participate in T cell contraction are less well characterized. A number of mechanisms have been reported (and recently compiled in a series of reviews [1]). These include regulation of cell death [2] and autophagy [3], upregulation of negative signaling molecules such as CTLA-4 [4] and PD-1 [5], metabolic amino-acid regulation [6], [7], control by T regulatory (Treg) cells [8] and Treg induction by dendritic cells [9], among many others. Thus, homeostasis of the immune system depends on a fine balance between immune cell induction and deactivation.

CD46 was first identified as a regulator of the complement cascade [10], [11], but has subsequently been shown to link innate immunity to acquired immunity. Its activation promotes T cell activation and differentiation. Costimulation of TCR with CD46 leads to increased T cell proliferation [12], and affects T cell morphology [13] and polarity [14]. Furthermore, CD46 activation leads to Tr1 Treg differentiation [15]. This was characterized by secretion of high amounts of IL-10 [15] and granzyme B [16]. Interestingly, a recent report demonstrates that CD46 can in fact switch T cell differentiation from a Th1 to a Tr1 phenotype, depending on IL-2 concentrations present in the milieu [17]. This underlines the importance of the plasticity of CD46 in controlling T cell activation. We have previously shown that Tr1 differentiation is altered in patients with multiple sclerosis (MS). IL-10 secretion upon CD3/CD46 costimulation was impaired in T cells from ∼50% of patients with MS [18], [19]. The lack of Tr1 differentiation in MS was recently confirmed by another study [20] and in a primate model of MS [21], and the dysregulation of CD46 pathways in T cells was recently described in patients with asthma [22] and with rheumatoid arthritis [17]. The role of CD46 in human diseases highlights its importance in controlling T cell activation, and further underlines the need to understand its regulation and the molecular mechanisms responsible for its functions.

CD46 is a type I membrane protein expressed in all nucleated human cells. Its isoforms, products of alternative splicing, have four complement control repeats (CCR) at the N-terminus, followed by a heavily glycosylated region rich in serine, threonine and proline, a transmembrane segment, and one of two short cytoplasmic tails termed Cyt1 and Cyt2 [23]. Both tails can transmit signals [24], [25]. Most cell types co-express Cyt1 and Cyt2 except for brain and kidney cells, which predominantly express Cyt2 [26], and their function is mostly unknown. As mice do not express CD46 except for testis, we initially studied their role in inflammation in a CD46 transgenic mouse model of T cell-dependent contact hypersensitivity. We reported that CD46-Cyt1 inhibits inflammatory responses, whereas Cyt2 augments inflammation [27]. We also demonstrated that CD3/CD46 coactivated T cells from MS patients have higher levels of CD46-Cyt2 mRNA compared to activated T cells from healthy donors [19]. This suggests that the higher level of CD46-Cyt2 transcript resulting from CD46 engagement in MS patients may influence their T cell responses. Recently CD46 was shown to be a substrate for the presenilin/γ-secretase (P/γS). Upon infection by pathogenic *Neisseria*, epithelial cell CD46 was sequentially cleaved by MMP and P/γS [28]. MMP cleavage releases a soluble ectodomain and a C-Terminal Fragment (CTF) consisting of the transmembrane region and cytoplasmic tail. P/γS then cleaves the CTF, releasing the Cyt1 and Cyt2 tails into the cytosol. Whether the Cyt1 and/or Cyt2 ICDs have biological activity is currently unknown.

Herein, we investigated the regulation of CD46 expression upon T cell activation and tested the hypothesis that P/γS modulates the function of CD46-Cyt1 and CD46-Cyt2 on immune function. We demonstrate a novel function of CD46 in terminating T cell activation. We first present evidence of CD46 processing in human primary T cells. We show that CD46 is cleaved by MMP in CD46-coactivated human T cells. Furthermore, our data illustrate that Cyt1 and Cyt2 levels fluctuate dynamically during T cell stimulation. CD28 costimulation results in an increase in Cyt1 and Cyt2 expression, suggestive of a crosstalk between CD46 and CD28. However, upon CD46 coactivation, CD46 cytoplasmic isoforms were temporally downregulated. Cyt1 expression decreased transiently, whereas Cyt2 expression increased then strongly decreased. Addition of P/γS enzymatic complex inhibitors impaired CD46 tail downregulation. We demonstrate the requirement of CD46 CTF processing in immune regulation by two approaches. First, we expressed uncleavable mutant CD46 Cyt1 and Cyt2 CTF constructs (hereafter called CTF1 and CTF2, respectively) in primary human T cells. Expression of wild-type (wt) CTF1 promoted T cell proliferation, CD25 expression and IL-10 secretion, whereas expression of uncleavable CTF1 (UNCL.F1) abrogated T cell activation, demonstrating that cleavage of Cyt1 is required for its function. Expression of wt CTF2 decreased IFNγ secretion, while expression of uncleavable CTF2 (UNCL.F2) enhanced T cell proliferation, increased CD25 expression and IFNγ secretion, indicating that Cyt2 cleavage acts as an inhibitory signal for T cell activation. Second, we expressed CD19 ectodomain-CD46 CTF fusion proteins in primary T cells. Triggering of Cyt1 or Cyt2 by CD19 ligation led to similar conclusions in terms of cytokine production and proliferation. Taken together, our data indicate that processing of CD46 tails is required to first promote T cell activation followed by signals resulting in T cell inhibition, demonstrating the unexpected role of CD46 in turning off its own activation in a negative feedback loop. These data suggest that the timely activity of P/γS on the two CD46 isoforms provides a molecular rheostat for regulating T cell activation.

## Results

### CD46 is cleaved by a metalloproteinase upon T cell activation

**Figure 1:**
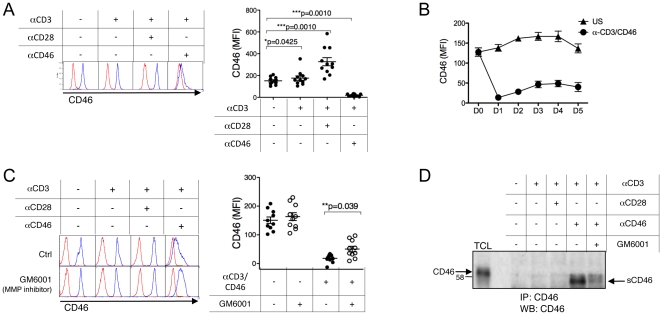
CD46 ectodomain is processed by MMPs upon T cell activation. (**A**) CD4^+^ T cells were left unstimulated, or stimulated as indicated with immobilized anti-CD3, anti-CD3/CD28 or anti-CD3/CD46 for 48 hrs. The expression of CD46 at the cell surface was examined by flow cytometry. The results obtained for one donor are shown. The normalized MFI for CD46 (Δ to the isotype control) are plotted for the different donors analyzed (mean ± SEM, n = 12). All data were analyzed using the Wilcoxon test, a non-parametric paired t-test that does not assume Gaussian distribution. (**B**) CD4^+^ T cells were activated with immobilized anti-CD3/CD46 antibodies or left unstimulated (US) for several days and CD46 surface expression monitored daily. (**C**) CD4^+^ T cells were activated with immobilized antibodies as indicated in presence of GM6001, a broad metalloproteinase inhibitor (10 µM), or DMSO as a control. After 2 days, the cell surface expression of CD46 was determined by flow cytometry. The representative plots obtained for one donor are shown, and the normalized data obtained for the different donors (n = 10) are shown on the right panels. (**D**) The presence of sCD46 in the cell culture supernatants of activated T cells, as indicated, was determined after CD46 immunoprecipitation and western-blot analysis. TCL =  Total Cell Lysate, as a control for membrane CD46. Representative of 2 experiments.

We first assessed whether activating primary human T cells via CD46 could modulate its surface expression. Purified human CD4^+^ primary T cells were activated by immobilized anti-CD3, anti-CD3 and anti-CD28 (anti-CD3/CD28), or anti-CD3 and anti-CD46 antibodies (CD3/CD46) for 2 days. The presence of CD46 ectodomain on T cells was monitored by flow cytometry. CD3 and CD3/CD28 costimulation led to an increase in surface CD46 levels (Figure 1A). In contrast, CD3/CD46 stimulation resulted in a loss of surface CD46. Reduced levels of surface CD46 were observed up to 5 days post-activation (Figure 1B).

In order to determine whether MMPs are involved in the downregulation of surface CD46, we cultured T cells in presence of GM6001, a broad MMP inhibitor. Activating T cells with CD3/CD46 in the presence of GM6001 partly restored surface CD46 levels (Figure 1C). Moreover, using an ectodomain-specific antibody, we were able to immunoprecipitate CD46 from supernatants of CD3/CD46 stimulated cells, but not from supernatants of unstimulated cells, or cells stimulated with CD3 or CD3/CD28 (Figure 1D). The slightly lower molecular weight of soluble CD46 compared to membrane CD46 is the size predicted for the ectodomain released by MMP. Furthermore, addition of GM6001 decreased the levels of soluble CD46 in the supernatants of CD3/CD46 activated T cells. Hence, T cell activation via CD46 causes its ectodomain to be released from the membrane, and MMP cleavage is responsible at least in part for this shedding. We next determined whether addition of MMP inhibitor could modulate CD46 function and notably IL-10 production. Addition of GM6001 slightly increased the proliferation of CD46-activated T cells. However, it significantly inhibited IL-10 production (Figure S1). These data suggest that CD46 processing may be required for IL-10 production by CD46-activated T cells.

### P/γS causes fluctuations in the levels of the two CD46 cytoplasmic tails

**Figure 2:**
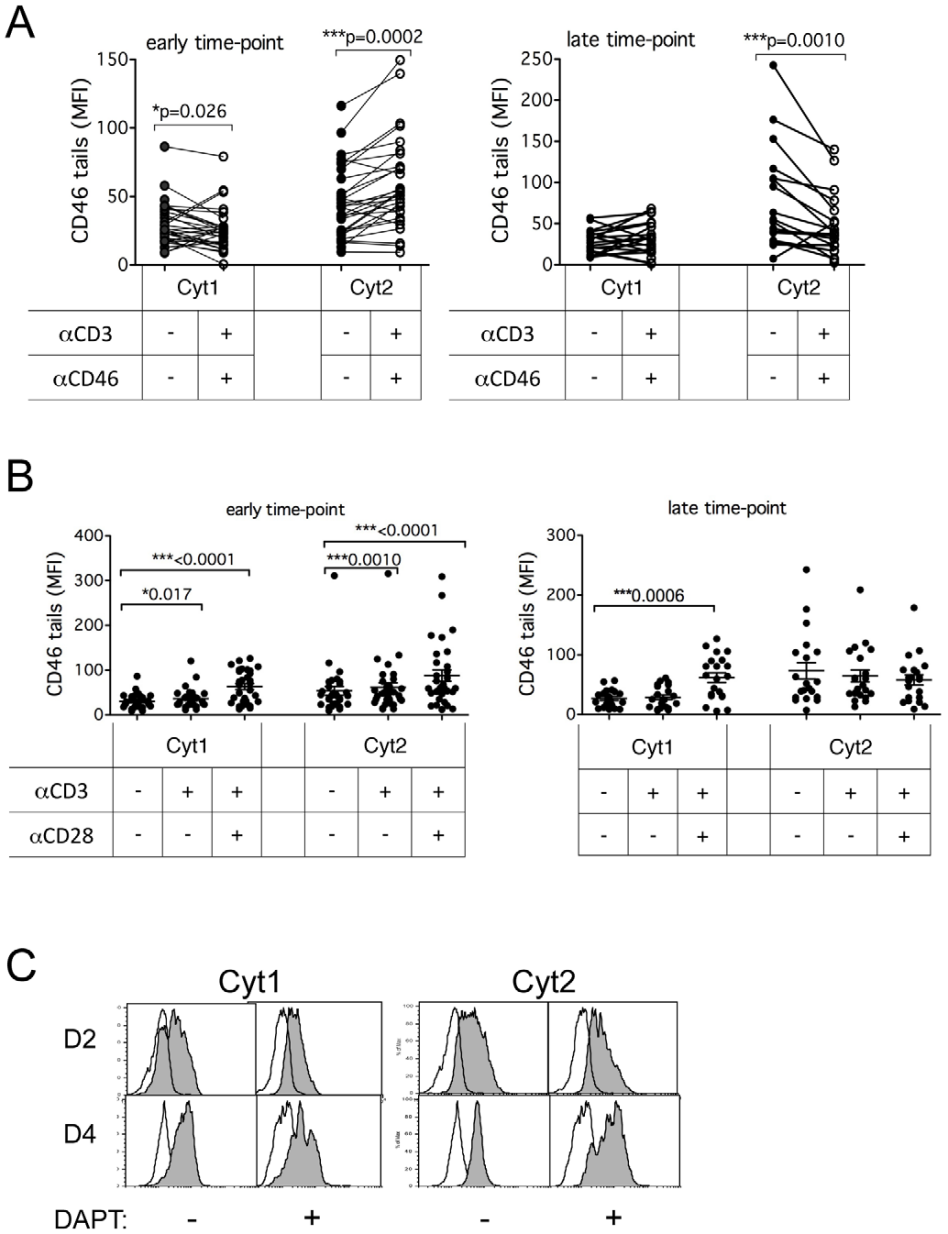
CD46 cytoplasmic tails are regulated by P/γS upon T cell activation. Purified CD4^+^ T cells were left unstimulated or stimulated as indicated by immobilized anti-CD3/CD46 (**A**), anti-CD3 or anti-CD3/CD28 (**B**), for 28–40 hrs (early time point) and 96–120 hrs (late time point). The expression of the two cytoplasmic tails of CD46 was determined by intracellular staining (0.1% saponin) using specific Cyt1 or Cyt2 monoclonal antibodies. The samples were analyzed using the Wilcoxon test, a paired test that does not assume Gaussian variation. The means ± SEM are shown. (**C**) CD4^+^ T cells were stimulated by immobilized anti-CD3/CD46 antibodies for 2 or 4 days in presence of DAPT (10 µM), a P/γS inhibitor, or of DMSO as control. The expression of Cyt1 and Cyt2 was then analyzed by flow cytometry. There was an increased expression of Cyt1 at D2 and increased Cyt2 levels at D4 in presence of DAPT. Representative of four experiments.

We next addressed the possibilities of further downstream processing of CD46 cytoplasmic tails in primary human CD4^+^ T cells. CD4^+^ primary T cells were activated by anti-CD3/CD46 antibodies for ∼28–40 hrs (early time point) or 96–120 hrs (late time point). CD46 tails Cyt1 and Cyt2 were monitored by flow cytometry using tail-specific monoclonal antibodies [29]. At the early time point, Cyt1 levels were reduced in stimulated cells compared to unstimulated cells (p = 0.026), whereas Cyt2 levels were significantly increased (p = 0.0002; Figure 2A and Figure S2). At the late time point, Cyt1 levels in stimulated and unstimulated cells were equivalent, whereas Cyt2 levels were significantly lower than those in unstimulated cells (Figure 2A and Figure S2). Stimulating cells with CD3 or CD3/CD28 antibodies, i.e., without CD46 ligation, slightly increased cytoplasmic Cyt1 at the early time point, with CD3/CD28 having the most dramatic effect (p = 0.0001; Figure 2B). Cyt1 levels remained elevated at the late time point, but only in CD3/CD28 stimulated cells (p = 0.0006). In contrast, CD3 and CD3/CD28 stimulated cells had increased Cyt2 levels only at the early time point, with CD3/CD28 stimulation having again the most dramatic effect (p = 0.0001). These data demonstrate that T cell costimulation by CD46 but also by CD28 regulates the levels of expression of CD46 cytoplasmic tails, and suggest a crosstalk between these two costimulatory molecules.

We next investigated whether P/γS is involved in regulating Cyt1 and Cyt2 tail levels. CD4^+^ T cells were activated by immobilized anti-CD3/CD46 antibodies in the presence of DAPT, a P/γS pharmacological inhibitor, or of DMSO as a control, for 2 or 4 days, and Cyt1 and Cyt2 levels were analyzed by flow cytometry using tail-specific monoclonal antibodies. A representative experiment with one set of donor cells is presented in Figure 2C. Both Cyt1 and Cyt2 levels were increased in the presence of DAPT, compared to cells stimulated in the absence of DAPT (Figure 2C). An increased expression of Cyt1 was detected at day 2 while increased expression of Cyt2 was detected at day 4. Similar results were obtained when the experiment was repeated with addition of L-685,458, another P/γS inhibitor (Figure S3). These inhibitors had no effect on the levels of surface CD46 (not shown). These data suggest that P/γS activity modulates the levels of Cyt1 and Cyt2 isoforms in T cells, in a time-dependent fashion.

### IL-2 has no significant effects on CD46 expression

**Figure 3:**
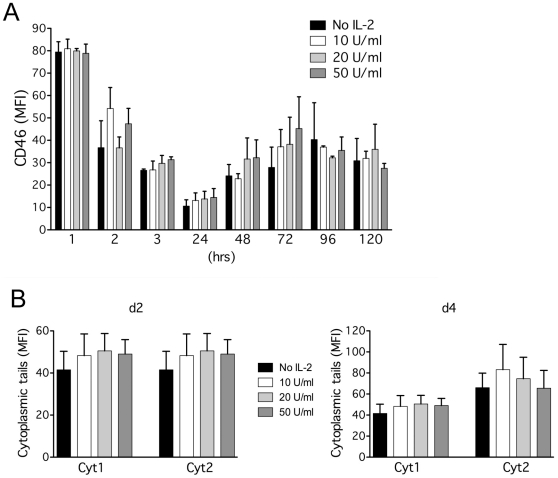
IL-2 has no significant effect on CD46 expression. Purified CD4^+^ T cells were stimulated by immobilized anti-CD3/CD46 with increasing concentrations of rhIL-2, as indicated, for various lengths of time. Expression of cell surface CD46 (**A**) and of intracellular Cyt1 or Cyt2 (**B**) was monitored by flow cytometry. The means ± SEM are shown (n = 5). The samples were analyzed using the Wilcoxon test, a paired test that does not assume Gaussian variation. A slight increase in expression of CD46 cytoplasmic isoforms was observed but no statistical differences were obtained.

CD46 costimulation drives Tr1 differentiation in presence of IL-2 [15], [17]. Hence, we assessed whether IL-2 modulates CD46 expression. CD4^+^ T cells were activated with anti-CD3/CD46 antibodies in presence of increasing doses of IL-2. Expression of cell surface CD46 and of its cytoplasmic isoforms were monitored overtime by flow cytometry (Figure 3). No significant effect was observed for surface CD46 expression (Figure 3A). Although there was a small increase in Cyt1 and Cyt2 expression in presence of IL-2, it was not significant (Figure 3B). Hence, IL-2 did not seem to considerably affect CD46 expression.

### The expression of the two CD46 CTFs differently affects T cell responses

**Figure 4:**
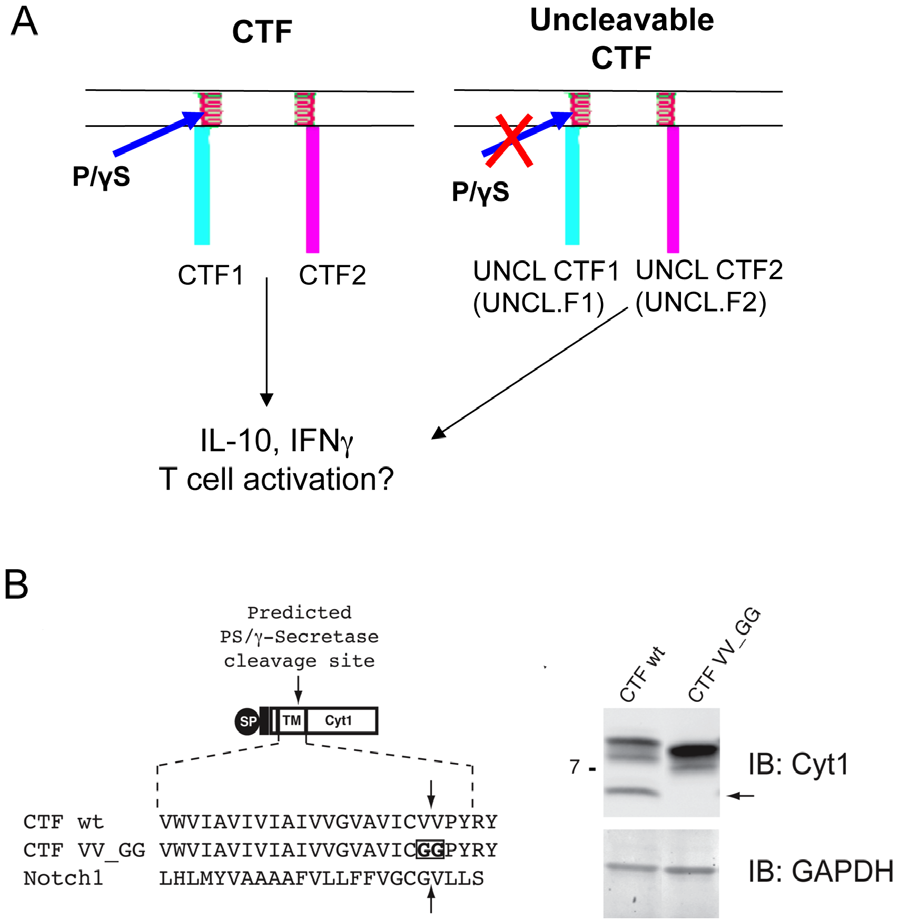
CTF constructs used in this study. (**A**) Schematic representation of the proteins encoded by the different CTF plasmids used in this study. Plasmids encoding the CTF portion of either Cyt1 (CTF1) or Cyt2 (CTF2) as well as mutants rendered uncleavable by the P/γS (UNCL.F1 and UNCL.F2) are represented. (**B**) Uncleavable CTF constructs. Left panel: Top diagram shows wt and mutant Cyt1 tail constructs. SP: heterologous signal peptide; black box: peptide linker; unlabelled white box: short segment of CD46 ectodomain; TM: CD46 transmembrane region; Cyt1 (or Cyt2 for CTF2 constructs) cytoplasmic tail. Lower diagram shows amino-acid sequence of the wt TM and uncleavable mutant TMs with amino-acid substitution (boxed residues). The Notch1 TM is shown for comparison. Downward arrow points to predicted P/γS cleavage site for CD46; upward arrow to known cleavage site for Notch1. The right panel shows the immunoblot of CHO cells expressing wt (CTF) and mutant (CTF VV_GG). Arrow indicates the 6 kD Cyt1 peptide release by P/γS cleavage. GAPDH =  loading control.

**Figure 5:**
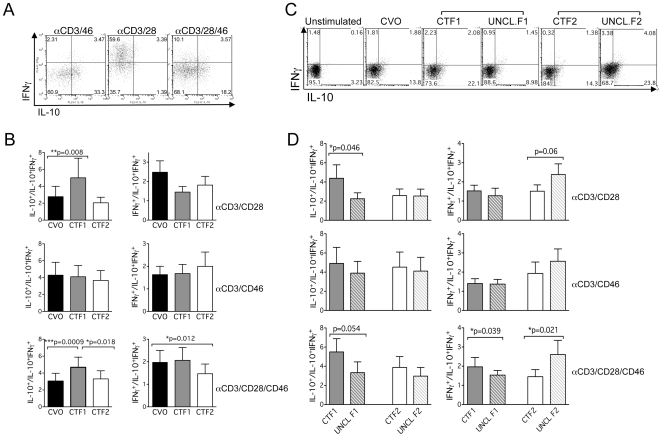
Cleavage of CTF-Cyt1 and CTF-Cyt2 differently controls IL-10 and IFNγ production. (**A**) Activation of transfected (with a control plasmid) primary CD4^+^ T cells results in normal differentiation. Twenty-four hours post transfection by Amaxa/Lonza with the empty vector control (CVO), CD4^+^ T cells were stimulated by immobilized anti-CD3/CD46, anti-CD3/CD28 or anti-CD3/CD28/CD46 monoclonal antibodies for 72 hrs. The secretions of IL-10 and IFNγ were assessed by secretion assays (Miltenyi). CD46 costimulation induces mainly IL-10 production, while CD28 coactivation induces more IFNγ. CD28/CD46 coligation also induces Tr1/IL-10 secretion (as described by [15]). (**B**) CD4^+^ T cells were transfected with the control plasmid (CVO) or encoding CTF-Cyt1 (CTF1) or CTF-Cyt2 (CTF2) fragments. Twenty-four hours post transfection, CD4^+^ T cells were stimulated by immobilized anti-CD3/CD28, anti-CD3/CD46 or anti-CD3/CD28/CD46 monoclonal antibodies, as indicated. After 3 days, the secretions of IL-10 and IFNγ were assessed by secretion assays (Miltenyi). The proportions of IL-10^+^/IL-10^+^IFNγ^+^ and of IFNγ^+^/IL-10^+^IFNγ^+^ secreting cells for the multiple experiments performed are represented. The means ± SEM are shown (n = 10). All data were analyzed using the Wilcoxon test, a non-parametric paired t-test that does not assume Gaussian distribution. (**C**) CD4^+^ T cells were transfected with the plasmids encoding CTF-Cyt1 (CTF1) or CTF-Cyt2 (CTF2) or with the uncleavable CTF1 or CTF2 (UNCL.F1 and UNCL.F2) before analysis of IL-10 and IFNγ production. The data obtained for one donor is shown upon CD3/CD28/CD46 activation. (**D**) The proportions of IL-10^+^/IL-10^+^IFNγ^+^ and of IFNγ^+^/IL-10^+^IFNγ^+^ secreting cells for the multiple experiments performed are represented (n = 7).

To further investigate the role of P/γS processing of CD46 in T cell functions, we next expressed the Cyt1 or Cyt2 CTF in primary T cells. These constructs consist of the membrane-spanning segment of CD46 and either the Cyt1 or Cyt2 cytosolic tail. Hereafter, these constructs are named CTF1 (containing the Cyt1 tail) or CTF2 (containing the Cyt2 tail) (Figure 4). We first checked that primary T cells transfected with empty vector responded normally to CD46 and CD28 co-stimulation. Indeed, these cells produced high levels of IL-10 and low levels of IFNγ when activated by CD46 antibodies, and higher levels of IFNγ when activated by CD28 antibodies. As reported, cells activated with CD28/CD46 produced more IL-10 than those activated by CD28 alone (Figure 5A and [15]). Cells were then transfected with plasmids encoding CTF1, CTF2 or with empty vector (CVO). Twenty-four hours post-transfection, the cells were activated with anti-CD3/CD28 or anti-CD3/CD46, or with anti-CD3/CD28/CD46, and after 4 days, IL-10 and IFNγ secretion was assessed by flow cytometry. We determined the proportion of IL-10^+^, IL-10^+^IFNγ^+^ and IFNγ^+^ secreting cells after expression of each construct and calculated the proportion of IL-10^+^/IL-10^+^IFNγ^+^ and of IFNγ^+^/IL-10^+^IFNγ^+^ to detect the potential effects of the CTFs on specific cytokine production (Figure 5B). Activation of CTF1-expressing cells by CD3/CD28 and CD3/CD28/CD46 increased the proportion of IL-10-secreting cells in the population compared to cells transfected by the empty vector (p = 0.008), and slightly decreased the proportion of IFNγ-secreting cells but only upon CD3/CD28 activation. CTF2 expression led to a significant decrease in IFNγ-secreting cells (p = 0.012) but had no significant effect on IL-10-secreting cells (Figure 5B,C). CD3/CD46-activated T cells remained mainly insensitive to CTF expression. This is likely due to the dominant stimulation of the endogenous CD46. However, as CTF expression could modulate the response of the cells coactivated by CD28/CD46, this suggests an effect of the CTF on the CD28 pathway, independently of the endogenous CD46, supporting the hypothesis of a crosstalk between CD28 and CD46.

Next, we transfected primary T cells with mutants of CTF1 and CTF2 that cannot be cleaved by P/γS (UNCL.F1 or UNCL.F2, respectively) (Figure 4). A representative experiment from one set of transfected donor cells upon CD3/CD8/CD46 activation is shown in Figure 5C. UNCL.F1-expressing cells secreted ∼50% less IL-10 and slightly less IFNγ than CTF1-expressing cells. An increase in IFNγ production was observed for UNCL.F2-expressing cells compared to CTF2-expressing cells. Although UNCL.F2-expressing cells secreted more IL-10 than CTF2-expressing cells, the proportion of IL-10^+^/IL-10^+^IFNγ^+^secreting-cells was unaffected. The proportion of IL-10^+^ and IFNγ^+^ only secreting cells upon the different conditions of stimulation obtained for 7 independent donors is also represented in Figure 5D. Inhibition of CTF1 cleavage abrogated the increase in IL-10^+^ cells and had a weaker effect on IFNγ-secretion. Inhibition of CTF2 cleavage enhanced the percentage of IFNγ-secreting cells.

**Figure 6:**
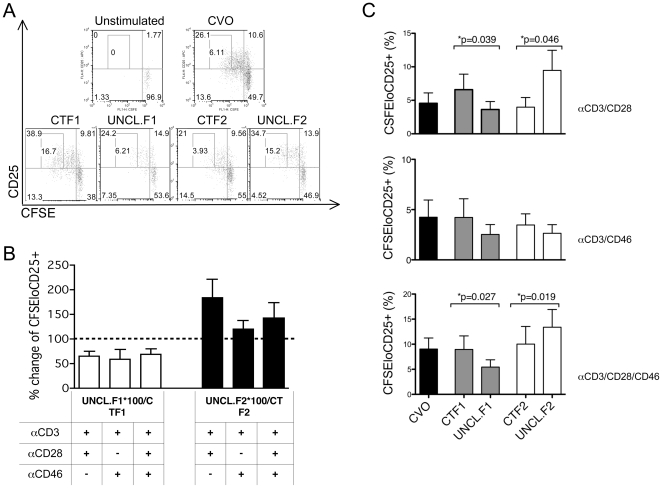
Cleavage of CTF-Cyt1 and CTF-Cyt2 differently controls T cell proliferation and CD25 expression. CD4^+^ T cells were transfected with the plasmid encoding CTF-Cyt1 (CTF1) or CTF-Cyt2 (CTF2), or the uncleavable fragments (UNCL.F1and UNCL.F2). Twenty-four hours post transfection, CD4^+^ T cells were labeled with CFSE and stimulated with immobilized antibodies for 4 days. Proliferation and CD25 expression was determined by flow cytometry. (**A**) The data obtained upon CD3/CD28 activation for one donor is shown. (**B**) The percentage of change in CFSEloCD25^+^ cells upon expression of the uncleavable constructs compared to the cleavable ones upon the different conditions of stimulation is represented as an average of several independent experiments with different donors (n = 7). (**C**) The average % of CFSEloCD25^+^ cells (inner gate in (**A**)) obtained in the different experiments performed is also represented.

We next assessed T cell activation levels by monitoring CD25 expression and proliferation in primary T cells expressing wild-type and uncleavable CTFs. Transfected T cells were labeled with CFSE, then activated with anti-CD3/CD46, anti-CD3/CD28 or anti-CD3/CD28/CD46 antibodies, and CD25 expression and proliferation were assessed by flow cytometry. A representative experiment with one set of transfected donor T cells upon CD3/CD28 activation is presented in Figure 6. CTF1-expressing cells had higher levels of CD25 and proliferated more than control cells. Expression of UNCL.F1 abrogated this effect (Figure 6A). CTF2-expressing cells proliferated similarly than control cells. However, expression of UNCL.F2 led to a strong increase in proliferation rate and CD25 expression, compared to CFT2 expressing cells, as shown by the increased percentage of CFSEloCD25^+^ cells (inner gate, Figure 6A). The percentage of changes in CFSEloCD25^+^ cells upon expression of the uncleavable constructs compared to cleavable ones obtained for 7 independent experiments are also represented in Figure 6B. The % of CFSEloCD25^+^ cells obtained for the different experiments are represented in Figure 6C. UNCL.F1-expressing cells had significant reduced levels of CFSEloCD25^+^ cells than CTF1-expresing cells, while expression of UNCL.F2 led to a significant increase in CFSEloCD25^+^ cells. This indicates that processing of the two isoforms regulated cell activation, albeit antagonistically. CTF1 cleavage was necessary to boost T cell activation, while CTF2 cleavage resulted in T cell inhibition.

### Specific triggering of CD46 cytoplasmic tail results in differential T cell activation profile

**Figure 7:**
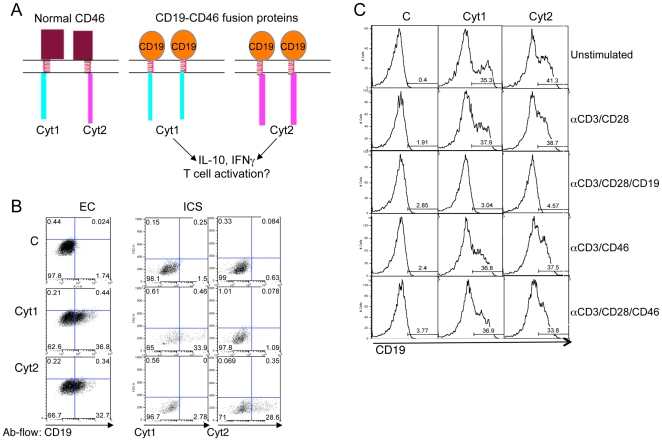
CD19-CTF fusion proteins used in this study. (**A**) Schematic representation of the proteins encoded by the different fusion plasmids used in this study. Plasmids encoding the CD19 ectodomain fused to either CTF-Cyt1 or CTF-Cyt2 was constructed. (**B**) Chimeric protein expression in HEK293 cells. HEK cells were transfected by Fugene with pcDNA3 plasmids (control (C), CD19-Cyt1 (Cyt1) or CD19-Cyt2 (Cyt2)). Twenty-four hours later, the expression of CD19, Cyt1 and Cyt2 was assessed by flow cytometry using anti-CD19-FITC and monoclonal antibodies specific of each isoform. (**C**) CD4^+^ T cells were transfected with the control pcDNA3 plasmid (C) or encoding the fusion proteins consisting of the extracellular domain of CD19 and CTF of either Cyt1 or Cyt2 and activated as indicated. Twenty-four hours later, CD19 expression was determined by flow cytometry. Representative of three experiments.

We next designed fusion constructs that would allow us to directly and specifically trigger Cyt1 or Cyt2 without affecting endogenous CD46. We constructed chimeric molecules consisting of the extracellular domain of CD19, a B cell marker, fused to CTF1 or CTF2 (Figure 7A). These constructs contain the MMP cleavage domain, and they were named CD19-Cyt1, or CD19-Cyt2. These constructs were first characterized for the correct expression of the chimeric proteins by transfecting them into HEK293 cells and examining fusion protein expression by flow cytometry using antibodies to CD19 ectodomain, Cyt1 and Cyt2 (Figure 7B). Approximately 40% of cells transfected with pcDNA3-CD19-Cyt1 expressed CD19 and high levels of Cyt1 while CD19 and high expression level of Cyt2 was only observed in pcDNA3-CD19-Cyt2 transfected cells. Expression in primary T cells also resulted in ∼40% expression of CD19 (Figure 7C). We assessed the functionality of the MMP cleavage site in these fusions. Transfected T cells expressing fusion constructs were left unstimulated, or were activated with immobilized anti-CD3/CD28 antibodies in presence or absence of immobilized anti-CD19 to specifically trigger the fusion proteins, or activated with anti-CD3/CD46, or anti-CD3/CD28/CD46 antibodies. Ligation of CD19 strongly decreased surface levels of CD19 (Figure 7C). This reduced CD19 staining was not due to the masking of the epitope by detached (and pre-immobilized) CD19 antibodies, as the cells did not react with FITC-anti-mouse IgG when harvested from culture (data not shown). These chimeric constructs therefore contain a functional MMP cleavage site.

**Figure 8:**
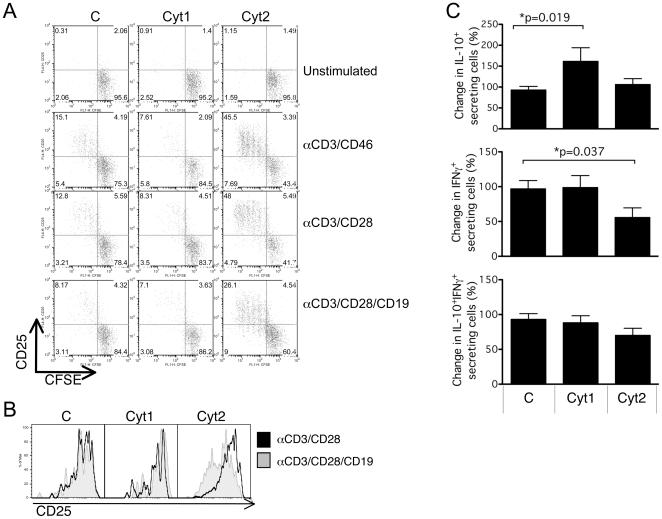
Specific triggering of Cyt1 and Cyt2 differently controls T cell activation. (**A**) CD4^+^ T cells were transfected with the control pcDNA3 plasmid (C) or encoding the CD19-CTF1 (Cyt1) or CD19-CTF2 (Cyt2) fusion proteins. Twenty-four hours post transfection, CD4^+^ T cells were labeled with CFSE. Labeled cells were then stimulated by immobilized anti-CD3/CD46, anti-CD3/CD28 or anti-CD3/CD28/CD19 monoclonal antibodies and proliferation as well as CD25 expression were determined by flow cytometry. (**B**) Proliferating cells were gated on CFSE-low cells and the expression of CD25 was determined. The black lines represent the staining obtained for CD3/CD28 activated cells, the shaded grey histograms represent the staining for the cells activated in presence of anti-CD19. Representative of three experiments. (**C**) Transfected cells were activated by anti-CD3/CD28 or anti-CD3/CD28/CD19 monoclonal antibodies. After 4 days, the secretions of IL-10 and IFNγ were assessed by secretion assays (Miltenyi). The changes in the percentages of IL-10^+^, IL-10^+^IFNγ^+^ and IFNγ^+^ secreting cells induced by CD19 ligation compared to secretion in absence of CD19 (100%) for the multiple experiments performed are represented (n = 9). Samples were analyzed using the Wilcoxon test.

We next determined whether specific activation of CD19-Cyt1 or CD19-Cyt2 could modulate T cell activation. Because coactivation by CD3/CD28 elicited a much stronger response than CD3 alone (not shown), we subsequently studied the effect of CD19 ligation on CD3/CD28 activation. Primary T cells expressing CD19-Cyt1, CD19-Cyt2 or empty vector were labeled with CFSE and then activated by immobilized anti-CD3/CD46, or by anti-CD3/CD28 antibodies in presence or absence of anti-CD19. After four days, proliferation and CD25 expression were assessed by flow cytometry. A representative experiment is shown in Figure 8A. Expression of CD19-Cyt1 resulted in a decrease of proliferation of the cells compared to cells transfected by the control vector, while CD19-Cyt2 expression by itself, without its ligation, increased proliferation. Ligation of CD19 had no significant effect of cells transfected by the control or expressing CD19-Cyt1. In contrast, a dramatic inhibition of proliferation was observed when CD19-Cyt2 was engaged. While no significant difference in CD25 expression was detected for the control cells and CD19-Cyt1 expressing cells, a strong decrease in CD25 expression was observed for CD19-Cyt2 expressing cells upon CD19 ligation (Figure 8A and B). These results therefore supported those observed using the CTF constructs. Expression of CD19-Cyt1 mainly mimicked UNCL.F1 expression while CD19-Cyt2 expression mainly reflects UNCL.F2 expression. However, specific activation of CD19-Cyt2 led to a striking inhibition of proliferation and CD25 expression, and indicated that Cyt2 differently modulates T cell activation depending on its triggering and processing.

We next determined the effect of Cyt1 and Cyt2 specific triggering on cytokine production. T cells expressing CD19-Cyt1 and CD19-Cyt2 were activated by CD3/CD28 or CD3/CD28/CD19, and cytokine production determined. The percentage of change in cytokine production upon CD19 ligation was calculated for IL-10^+^, IL-10^+^IFNγ^+^ and IFNγ^+^ cells. Co-activation of CD19-Cyt1 cells increased the percentage of cells producing IL-10 only (p = 0.019; Figure 8C). CD19-Cyt1 had no significant effect on the proportion of IL-10^+^IFNγ^+^ cells and on IFNγ^+^ secreting cells. In contrast, CD19 co-activation of CD19-Cyt2 cells significantly reduced IFNγ production (p = 0.037). Finally, CD19 co-activation of vector only control cells had no effect on either IL-10 or IFNγ production. These results corroborate the findings from CTF-expressing cells that altered the proportion of IL-10^+^ or IFNγ^+^-only producing cells.

### Specific triggering of CD19-Cyt2 results in increased CTLA-4 expression and dephosphorylation of LAT

**Figure 9:**
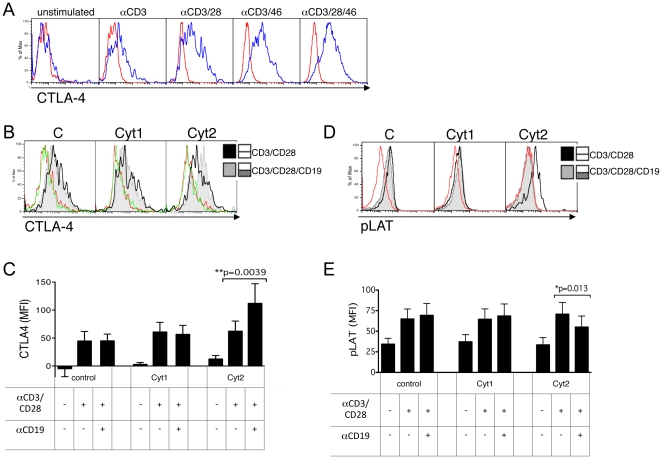
Specific Cyt2 triggering increases CTLA-4 expression and decreases LAT phosphorylation. (**A**) CTLA-4 expression was determined by flow cytometry on untransfected primary T cells activated as indicated for 4 days. (**B**) Twenty-four hours post transfection with pcDNA3 plasmids (control, CD19-Cyt1 or CD19-Cyt2), CD4^+^ T cells were stimulated with anti-CD3/CD28 (black plain line) or anti-CD3/CD28/CD19 (shaded grey area) monoclonal antibodies for 5 days. The expression of intracellular CTLA-4 was then assessed by flow cytometry. Red and green lines represent the staining obtained with the isotype controls for both types of activation. The average normalized CTLA-4 staining obtained for 5 experiments is represented in (**C**). Transfected cells were activated with anti-CD3/CD28 with or without CD19 and addition of a cross-linker for 5 min at 37°C. Activated cells were immediately fixed and then permeabilized. The presence of phospho-LAT was detected with an anti-pLAT by flow cytometry. Data representative of one donor are shown in (**D**). Red lines represent the staining obtained with the isotype controls. The average normalized pLAT staining obtained for 4 experiments is represented in (**E**).

In order to determine a possible mechanism for the inhibitory effect of Cyt2, we analyzed CTLA-4 levels, a potent co-inhibitory molecule for T cells [30]. As shown in Figure 9A, CTLA-4 expression is induced by activation of primary human T cells, and notably when CD46 is stimulated. In transfected cells, activation by CD19 had no effect on CD19-Cyt1 expressing cells and control cells. However, triggering of CD19-Cyt2 led to a strong increase in CTLA-4 expression, providing a possible mechanism for Cyt2 inhibitory role. The results obtained for one experiment is shown in Figure 9B, and the average data obtained for the different donors represented in Figure 9C.

CTLA-4 engagement decreases T cell activation by transmitting negative signals. Notably, it induces the dephosphorylation of proteins activated by TCR stimulation, such as LAT [31]. CD46 activation of T cells induces LAT phosphorylation [12]. Hence, we next determined the level of LAT phosphorylation upon activation of each tail. CD4^+^ T cells were transfected with the control plasmid or the plasmids encoding CD19-Cyt1 or CD19-Cyt2 and activated for 5 min in presence of anti-CD3/CD28 with or without CD19 ligation. The phosphorylation of LAT was analyzed by flow cytometry. The results obtained for one experiment are shown in Figure 9D, and the average data obtained for the different donors represented in Figure 9E. As expected, no difference was observed for the control cells when CD19 was activated. No significant effect was observed by the simultaneous triggering of CD19-Cyt1 and CD3/CD28. In contrast, CD19-Cyt2 costimulation induced a significant decrease in LAT phosphorylation, supporting the hypothesis of the role of Cyt2 in terminating T cell activation.

## Discussion

The outcome of T cell activation results from a computation of signals received by the T cells, ensuring first proper activation, followed by adequate termination of the immune response initiated [1]. Here, we demonstrate the ability of CD46, a major costimulatory molecule for human T cell activation [12], to provide both coactivation and termination signals, through the regulation of expression of its two cytoplasmic isoforms. We first demonstrate the regulation of CD46 processing. An MMP-dependent cleavage of CD46 ectodomain was initially observed at the cell surface of T cells when CD46 was ligated. The antibodies used for activation and labeling were different, and there was a clear effect of MMP inhibitor indicating that an MMP-dependent cleavage was at least partly involved. We also investigated whether CD46 activation induced its internalization by measuring intracellular levels of CD46 after intracellular staining and acid-stripping of the cells, as reported in [32] (data not shown). No significant increase in CD46 was detected in these conditions, suggesting that most CD46 was shed from the surface. This also correlated with the presence of soluble CD46 in the culture supernatants of coactivated T cells. Apoptosis of neuronal and epithelial cells also triggers CD46 MMP-dependent shedding [33], [34], indicating that several biological pathways use this mechanism of regulation. The loss of CD46 expression at the cell surface was followed by the downregulation of its cytoplasmic isoforms, although with a strikingly different time-course. Cyt1 was downregulated first, while Cyt2 downregulation was observed afterwards. The decrease in Cyt1 and Cyt2 expression could be partially restored in presence of P/γS inhibitors, in a timely fashion. This suggests that, upon T cell activation, this enzymatic complex cleaves at least a part of CD46 cytoplasmic tails. We were barely able to detect by western-blots these fragments in activated primary T cells (not shown). This may be due to the short half-life of these fragments once released in the cytosol and the limit in sensitivity of our assay with primary T cells. However, we could detect the decrease in full length CD46 upon its activation (not shown). Importantly, the processing by P/γS of both CD46 cytoplasmic isoforms has been observed upon binding of the pathogenic *Neisseria* bacteria, *N. gonorrhoeae* and *N. meningitidis*, to epithelial cells [28]. Hence, CD46 processing might be a general pathway initiated by its triggering, producing active ICD that will transduce signals.

Most importantly, we demonstrate herein the dual role of CD46 in regulating human T cell activation. Ten years ago, it was discovered that CD46 could act as a costimulatory molecule for human T cells [12] and few years later that it could drive Tr1 differentiation [15]. Our data now illustrate the role of CD46 in turning off T cell activation, providing a novel concept in the regulation of the immune response. This “yin and yang” ability of CD46 in regulating T cell activation is mediated by the P/γS-dependent processing of its two cytoplasmic isoforms. Activation of T cells by CD46 leads to Tr1 differentiation [15]. Our data indicate that Tr1 differentiation initiated by CD46 activation is mainly due to Cyt1, as its specific activation using the CD19-CD46 fusion protein promoted IL-10 secretion, previously characterized as a marker of CD46-induced Tr1 cell differentiation [15], [16]. An increase in Granzyme B, another hallmark of CD46-induced Tr1 cells [16] was also observed (data not shown). Importantly, the role of Cyt1 in IL-10 production was corroborated by the CTF constructs, as expression of CTF1 increased the proportion of IL-10-secreting cells. It also promoted T cell proliferation and CD25 expression. The cleavage of CTF1 was required for these functions, as they were abrogated by expression of the uncleavable CTF1 mutant. As CD3/CD28 activation induced a much stronger response than CD3 activation alone, we mainly studied the effects of CTF on CD3/CD28 or CD46 co-activated T cells. However, we were able to detect similar effects of the CTF constructs on CD3-activated T cells when CD3 induced sufficient activation levels (Figure S4). Expression of uncleavable CTF1 appears to block the cells in an unactivated state and fewer cells get activated. Expression of CD19-Cyt1 has the same effects – there are fewer cells capable of being activated. Hence, the decrease in proliferation of CD19-Cyt1 expressing cells, without its ligation, correlates with the results obtained by expressing UNCL.F1. The increase in proliferation and CD25 expression was not detected in CD19-activated cells expressing CD19-Cyt1 fusion protein. This may relate to the strength of activation of the cells, as the effect of CTF1 was only observed in CD3/CD28 co-activated T cells. The specific engagement of Cyt2 led to strikingly different effects. Expression of uncleavable CTF2 strongly promotes T cell activation. Similar results are obtained when CD19-Cyt2 is expressed. This suggests that Cyt2 can trigger signaling events independently of extracellular ligation, and participates in T cell activation at the early time point. Specific Cyt2 activation, via CD19 ligation, provokes its cleavage as demonstrated by the loss of CD19 expression upon its engagement, and results in lowered IFNγ secretion, as well as dramatically decreased proliferation and CD25 expression compared to CD3/CD28 activation in absence of CD19. Similarly, expression of an uncleavable CTF2 resulted in an increased IFNγ production, enhanced proliferation and CD25 levels. Furthermore, specific triggering of Cyt2 could enhance CTLA-4 level, a potent co-inhibitory molecule for T cells [30], providing a possible mechanism for Cyt2 inhibitory role. Indeed, we could demonstrate that specific Cyt2 triggering led to a decrease in LAT phosphorylation. Interestingly, CD46-activated T cells do not sustain proliferation over longer activation periods [35]. We have shown that gated CTLA-4^+^ cells exhibit a strong inhibition of proliferation when Cyt2 in engaged (Figure S5). Our data suggest an involvement of Cyt2 by upregulation of CTLA-4 and subsequent dephosphorylation. Together, these data and the results obtained using CTF2 clearly demonstrate that the lack of cleavage results in increased T cell activation. This illustrates that CTF2 processing is required to trigger inhibitory signals within activated T cells, in a negative feedback mechanism.

Importantly, while this manuscript was being written, it was reported that CD46 activation could switch T cells from a Th1 toward to Tr1 phenotype depending on IL-2 concentrations present in the milieu [17]. However, in our hands, we did not observe significant effects of increasing IL-2 concentrations on CD46 processing. Remarkably though, the authors found that expression of Cyt1 in Jurkat cells led to increased IL-10 secretion. Moreover, ligation of CD46 in γδ T cells that express more Cyt2 than Cyt1 (at least by PCR) results in a decrease in IFNγ expression. Our data corroborate these findings in primary CD4^+^ T cells, but they also demonstrate the role of CD46 isoforms in overall primary human T cell activation, and we provide evidence of the requirement of their processing in such functions. Importantly, both studies highlight the importance of the plasticity of CD46 in controlling T cell activation. While Kemper's group shows the ability of CD46 to switch cytokine production depending on IL-2 concentrations, we demonstrate that within the Tr1 differentiation conditions, CD46 acts as a rheostat for T cell activation. We first observed the loss of expression of Cyt1, while Cyt2 processing occurred later. This supports the notion that the initial regulation of CD46-Cyt1 induces IL-10 production and Tr1 differentiation, while the later Cyt2 processing results in switching off Tr1 cells, in a negative feedback mechanism. We had previously reported that CD46 cytoplasmic isoforms had differential roles in inflammation using transgenic mice expressing either isoform. In an *in vivo* model of contact hypersensitivity reaction to DNFB, CD46-Cyt1 had a potent anti-inflammatory role, while Cyt2 promoted inflammation [27]. Our data in human cells corroborate the anti-inflammatory role of Cyt1 through activation and Tr1 differentiation, and the pro-inflammatory role of Cyt2 may be explained by decreased IFNγ production [36], [37] and lack of Tr1 differentiation. Importantly, CD46 is defective in MS, as CD46-activation of both T cells and dendritic cells leads to a pro-inflammatory phenotype [19], [38]. Whilst CD46 activation induced Tr1 differentiation and IL-10 secretion in T cells from healthy donors, IL-10 production was decreased by T cells from MS patients [19], [20], [21], [39]. This was associated with an increased RNA expression of the Cyt2 isoform upon T cell activation [19]. Our data now reinforce the likely role of the abnormal Cyt1/Cyt2 ratio in the lack of Tr1 differentiation in patients with MS.

**Figure 10:**
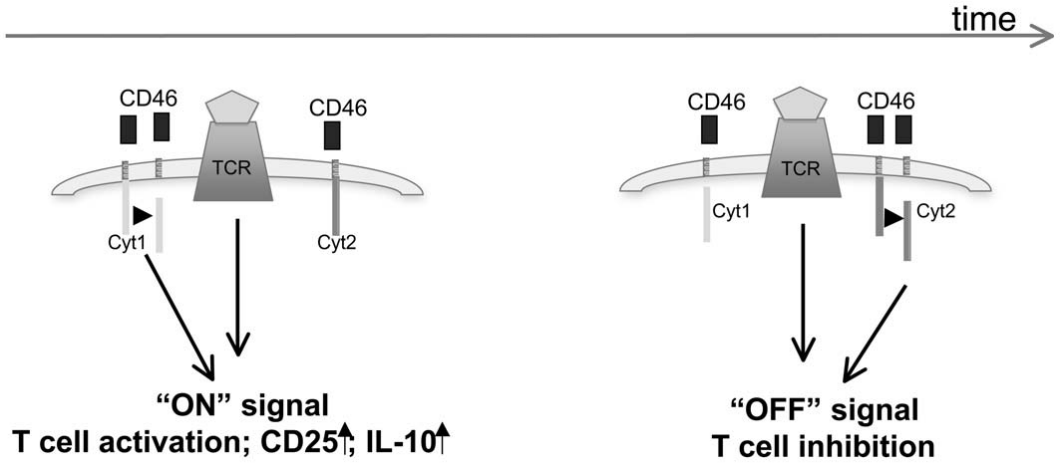
Proposed model of the yin and yang role of CD46 in human T cell activation. Upon CD3/CD46 activation, there is downregulation of Cyt1 expression and increase in Cyt2 expression at the early time point compared to unstimulated T cells. Cyt1 expression level then returns to the level of expression observed in unstimulated T cells, while the downregulation of Cyt2 expression occurs at the late time point. We propose that Cyt1 isoform is first cleaved by P/γS while there is an increase in Cyt2 expression. This results in T cell activation, increased proliferation and CD25 expression as well as IL-10 secretion. Later on, Cyt2 is processed by the P/γS, which induces a negative feedback mechanism and results in T cell inhibition, with lowered IFNγ secretion, decreased proliferation and CD25 expression and increased CTLA-4 expression.

Overall, our data suggest that CD46, through the temporal processing of its cytoplasmic tails, acts as a molecular rheostat for human T cells. We propose that, first, Cyt1 is cleaved which promotes T cell activation and IL-10 production, and that, later, Cyt2 cleavage sends a negative feedback message, turning off T cell activation, as summarized in the model depicted in Figure 10 and based on the data presented in Figure S6. Further elucidation of the transduction cascades initiated by CD46 isoforms will be required to fully understand their role in human T cell activation. The understanding of the mechanisms regulating their expression will provide a potent tool for immuno-therapies and will complete our growing knowledge on T cell activation and contraction in health and disease.

## Materials and Methods

### Cell purification and activation

PBMC were isolated by Ficoll-Hypaque density gradient centrifugation (Pharmacia LKB Biotechnology, Piscataway, NJ), from heparinized venous blood from healthy donors obtained after informed consent. Ethical approval was obtained from the Lothian Board Ethics Committee. CD4^+^ T cells were negatively isolated using magnetic beads (CD4 isolation kit II, Miltenyi Biotec, Auburn, CA, purification >90%). T cells were then cultured in culture wells pre-coated with anti-CD3 (OKT3, 5 µg/ml), anti-CD28 (CD28.2, 10 µg/ml), anti-CD46 (10 µg/ml) (20.6, kindly provided by Dr. Chantal Rabourdin-Combe, France), anti-CD19 (AbD Serotec, 10 µg/ml), or irrelevant IgG1 (Invitrogen, 10 µg/ml). Exogenous IL-2 (10 U/ml) was added to CD3/CD46 stimulated cells as previously described [15]. In some experiments, the P/γS inhibitors DAPT (N-[N-(3,5-Difluorophenacetyl-L-alanyl)]-S-phenylglycine t-Butyl Ester) or L-685,458 (Sigma-Aldrich) were added to the culture.

### Cell transfection

CD4^+^ T cells were negatively isolated (Miltenyi, purification >90%) and transfected (Amaxa/Lonza), following the manufacturer's instructions (U-14). HEK293 cells were transfected by Fugene (Roche). Twenty-four hours later, the expression of CD19, Cyt1 and Cyt2 was assessed by flow cytometry using CD19-PE (BD Biosciences), or specific monoclonal antibodies against Cyt1 or Cyt2 [29] (in 0.1% saponin).

### CTF constructs

The CTF expression constructs were made by subcloning PCR products amplified from CD46 pSecTag2/Hygro (Invitrogen) clones for Cyt1 and Cyt2 into pIRESneo (GenBank Accession no. U89673). PCR products were generated using primers CTF_IRES_EcoR1_F with IRES_BamHI_R_CTF1 for Cyt1 and CTF_IRES_EcoR1_F and IRES_BamHI_R_CTF2 for Cyt2. PCR products were digested with EcoR1 and BamH1 prior to cloning into pIRESneo. The predicted P/γS cleavage site was mutagenized with the VV_GG and VV_GG_rc primers using the QuikChange Lightning Site-Directed mutagenesis kit (Stratagene) according to the manufacturers instructions (Figure 4).

### Fusion proteins

The extracellular domain of CD19 was fused to CD46 CTF1 or CTF2. After total RNA extraction from HeLa and Raji cells, cDNAs were obtained using specific primers: AA3 for CD19, AA4 for Cyt-1 and AA5 for Cyt-2 (Table 1). The CD19 extracellular region was amplified by PCR with the primers AA1 and AA2 using PHUSION hot start polymerase (Finnzyme) with the addition of *HindIII* and *XbaI* restriction sites at the 5′ and 3′ ends, respectively. Cyt1 and Cyt2 transmembrane and intracytoplasmic domains were amplified with the pairs of primers AA3, AA4 and AA3, AA5 respectively. Amplified CD19, Cyt1 and Cyt2 cDNA were cloned in pBluescript II plasmid (Stratagene) and sequenced. Chimeric molecules containing CD19 extracellular region fused to the CTF domain of either CD46-Cyt1 or Cyt2 isoform were created by SOE-PCR [40], [41] using PHUSION hot start polymerase. For chimeric CD19-Cyt1, primer AA1 annealed at the 5′ end of CD19; primer AA4 annealed at the 3′ end of Cyt1 cDNA. Primers AA6 and AA7 were complementary to each other, overlapping at the respective fusion point of the two cDNA molecules. The chimeric cDNA was cloned into pcDNA3 vector (Invitrogen) using the *HindIII* and *XbaI* restriction sites introduced in the cDNA. The chimeric CD19-Cyt2 construct was similarly generated, using primers AA1, AA5, AA6 and AA7. All constructs were verified by sequencing.

**Table 1:** Primers used for cDNA cloning and generation of CTF and chimeric CD19-CD46 molecules^1^.

PRIMER NAME	SEQUENCE
**CTF CONSTRUCTS**	
CTF_IRES_EcoR1_F	CTAGAATTCCCACTGCTTACTGGCTTATCG
IRES_BamHI_R_CTF1	GATCGGATCCTCAGAGAGAAGTAAATTTTACTTCTCTGTGG
RES_BamHI_R_CTF2	GATCGGATCCTCAGCCTCTCTGCTCTGCTGGAG
VV_GG	GTTGGAGTTGCAGTAATTTGTGGTGGCCCGTACAGATATCTTCAAAG
VV_GG_rc	CTTTGAAGATATCTGTACGGGCCACCACAAATTACTGCAACTCCAAC
**FUSION PROTEINS**	
AA1	5′-CCCCCC**AAGCTT**AGTCTGACCACCATGCCACC-3′
AA2	5′-CCCCCC**TCTAGA**CTTCCAGCCACCAGTCCTCAG-3′
AA3	5′-CCCCCC**AAGCTT**GATGTTTGGGTCATTGCTGTG-3′
AA4	5′- CCCCCC**TCTAGA**TCAGAGAGAAGTAAATTTTACTTCTC-3′
AA5	5′-CCCCCC**TCTAGA**TCAGCCTCTCTGCTCTGCTG-3′
AA6	5′-CAGCAATGACCCAAACATCCTTCCAGCCACCAGTCCT-3′
AA7	5′-AGGACTGGTGGCTGGAAGGATGTTTGGGTCATTGCTG-3′

1 Bold letters indicate HindII and XbaI restriction sites. Overlapping sequences for generation of chimeric cDNA are underlined.

### Proliferation assay

Eighteen hours post transfection, T cells were labeled with CFSE after extensive washes with cold PBS, for 10 min at 37C. After quenching the reaction with cold 10%RPMI and further washes, the cells were seeded in 96-well plates precoated with immobilized antibodies as indicated. Four days later, the proliferation and CD25 expression (anti-CD25-APC) was assessed by flow cytometry.

### IFNγ and IL-10 secretion assays

Eighteen hours post transfection, T cells were seeded into 48-well culture plates (2.5×10^5^ cells per well) pre-coated with various antibodies. Four days later, the cells were harvested and the amounts of IL-10- and IFNγ-secreting cells were determined using the secretion assays from Miltenyi (IL-10-PE; IFNγ-APC).

### Flow cytometry

The expression level of CD46 ectodomain was assessed by flow cytometry with anti-CD46-FITC (BD Pharmingen). The expression level of Cyt1 or Cyt2 was performed by intracellular flow cytometry staining (with 0.1% saponin) using the specific monoclonal anti-Cyt1 or Cyt2 antibodies previously generated [29]. The relative expression to the staining with the isotype control was calculated by calculating the ΔMFI (MFI antibody stained - MFI control antibody).

### Detection of phospho-LAT

Transfected cells were activated with the appropriate Abs (CD3/CD28 with CD19 or irrelevant IgG1) and addition of a cross-linker for 5 min at 37°C [12]. Activated cells were immediately fixed (Cytofix, BD Biosciences) and then permeabilized (Perm buffer, BD Biosciences). The presence of phospho-LAT was detected with an anti-pLAT (pY171; BD Biosciences) and analyzed by flow cytometry.

### Statistical analyses

The groups were analyzed using the Graphpad Prism software. Data were analyzed using the Wilcoxon test, a non-parametric test that does not assume Gaussian variation. All p-values are two-tails and with a 95% confidence interval.

## Supporting Information

Figure S1 **Addition of the GM6001 metalloproteinase inhibitor inhibits IL-10 production by CD46-activated T cells.** Purified CD4^+^ T cells were left unstimulated, or stimulated by immobilized anti-CD3 or anti-CD3/CD46, as indicated, in presence of GM6001 or DMSO as control for 4 days. The proliferation was then assessed by thymidine incorporation, and the levels of IL-10 and IFNγ secreted in the culture supernatants were analyzed by ELISA.
(TIFF)

Figure S2 **Timely downregulation of expression of Cyt1 and Cyt2 upon T cell activation.** Purified CD4^+^ T cells were left unstimulated, or stimulated by immobilized anti-CD3/CD46, as indicated, for 28–40 hrs (early time point) and 96–120 hrs (late time point). The expression of the two cytoplasmic tails of CD46 was determined by intracellular staining (0.1% saponin) using specific anti-Cyt1 or Cyt2 monoclonal antibodies (blue line), or isotype control (red line). The data obtained for three different donors are shown.
(TIFF)

Figure S3 **Inhibition of P/γS increases the levels of Cyt1/Cyt2 expression.** CD4^+^ T cells were stimulated by immobilized anti-CD3/CD46 antibodies for 2 or 4 days in presence or absence of L-685,458, a P/γS inhibitor. The expression of Cyt1 and Cyt2 was then analyzed by flow cytometry. Addition of L-685,458 increases the levels of Cyt1 and Cyt2.
(TIFF)

Figure S4 **CTF expression can also alter the profile of cytokine produced and the proliferation of CD3-activated transfected T cells**. Twenty-four hours post transfection by Amaxa/Lonza with the different CTF constructs, CD4^+^ T cells were stimulated by immobilized anti-CD3 or anti-CD3/CD28 antibodies. (**A**) The secretions of IL-10 and IFNγ were assessed by secretion assays (Miltenyi). (**B**) Proliferation was assessed by flow cytometry (n = 8). In some experiments, anti-CD3 stimulation was very weak – hence we mainly studied the effects of expression of CTF in CD3/CD28 activated T cells. However, in the experiments where it induced T cell activation, we were then able to observe a similar effect of the CTF constructs.
(TIFF)

Figure S5 **Cyt2 inhibits proliferation of CTLA-4^+^ cells.** Twenty-four hours post transfection by Amaxa/Lonza with the different CD19-CD46 fusion proteins, CD4^+^ T cells were pre-labeled with CFSE and then stimulated by immobilized anti-CD3/CD28, anti-CD3/CD28/CD19 or anti-CD3/CD28/CD46 antibodies and CTLA-4 expression was determined after 4 days. The proliferation of CTLA-4^+^ gated cells is shown.
(TIFF)

Figure S6 **Kinetics of Cyt1 and Cyt2 expression upon CD46-coactivation.** Purified CD4^+^ T cells were left unstimulated (US), or stimulated by immobilized anti-CD3/CD46 (Stim), as indicated, for several days. The expression of the two cytoplasmic tails of CD46 was determined by intracellular staining using specific anti-Cyt1 or Cyt2 monoclonal antibodies (n = 5).
(TIFF)
