# Supplementary figures and images for: The Dynamic Processing of CD46 Intracellular Domains Provides a Molecular Rheostat for T Cell Activation
Source: PLoS One. 2011 Jan 19;6(1):e16287. doi: 10.1371/journal.pone.0016287 (PMC3023775; doi:10.1371/journal.pone.0016287)

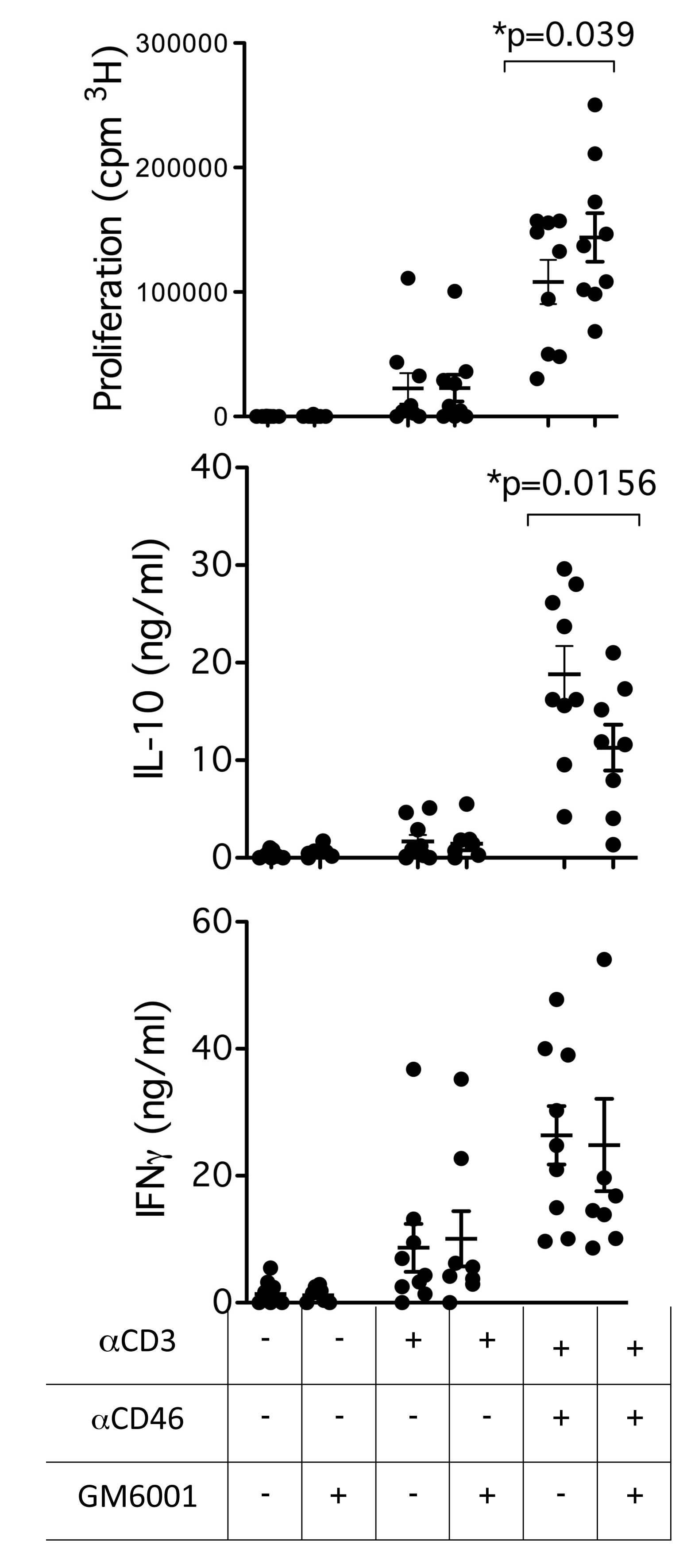

Supplement: Figure S1 — Addition of the GM6001 metalloproteinase inhibitor inhibits IL-10 production by CD46-activated T cells. Purified CD4+ T cells were left unstimulated, or stimulated by immobilized anti-CD3 or anti-CD3/CD46, as indicated, in presence of GM6001 or DMSO as control for 4 days. The proliferation was then assessed by thymidine incorporation, and the levels of IL-10 and IFNγ secreted in the culture supernatants were analyzed by ELISA. (TIFF) [file pone.0016287.s001.tif]

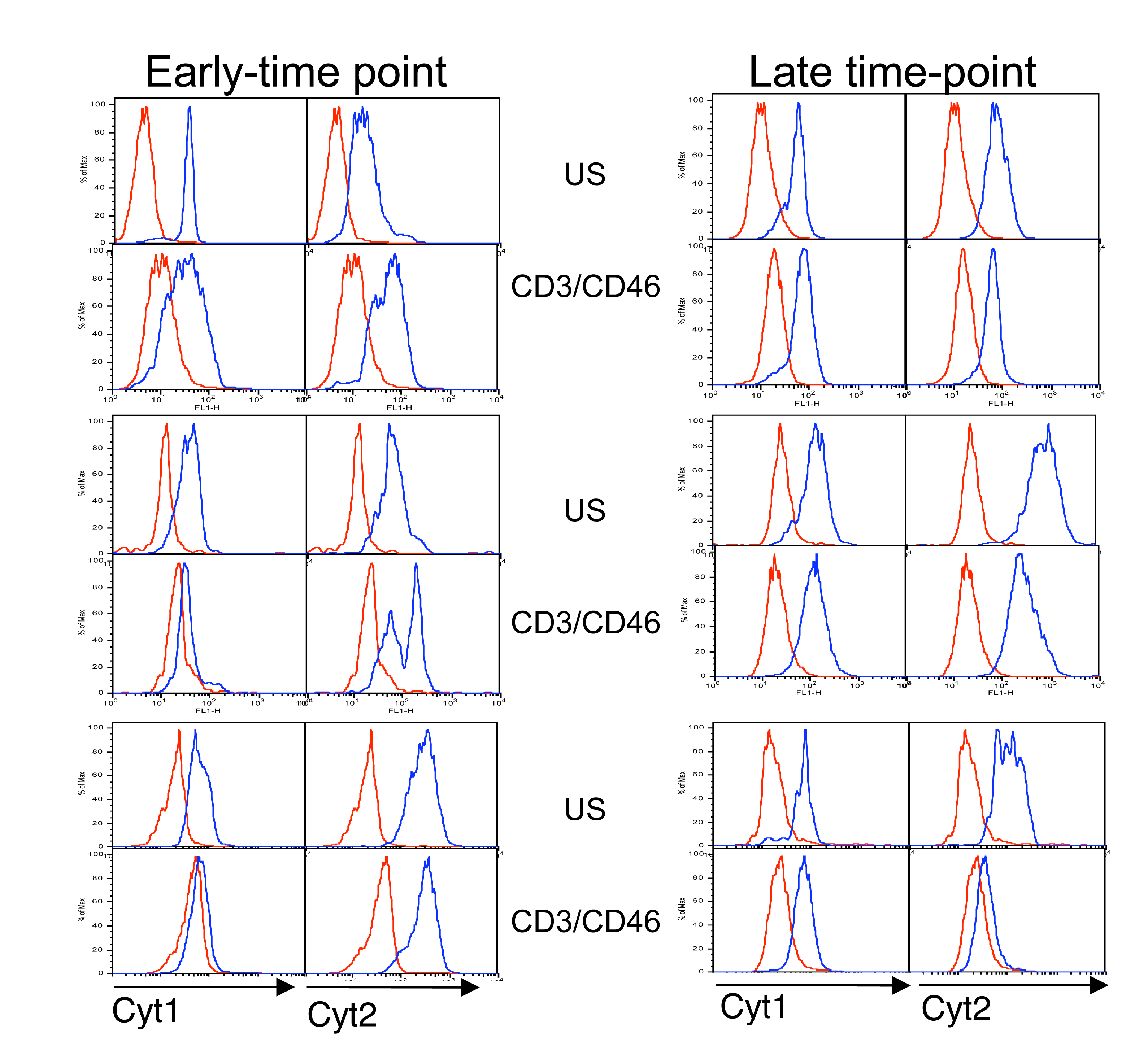

Supplement: Figure S2 — Timely downregulation of expression of Cyt1 and Cyt2 upon T cell activation. Purified CD4+ T cells were left unstimulated, or stimulated by immobilized anti-CD3/CD46, as indicated, for 28–40 hrs (early time point) and 96–120 hrs (late time point). The expression of the two cytoplasmic tails of CD46 was determined by intracellular staining (0.1% saponin) using specific anti-Cyt1 or Cyt2 monoclonal antibodies (blue line), or isotype control (red line). The data obtained for three different donors are shown. (TIFF) [file pone.0016287.s002.tif]

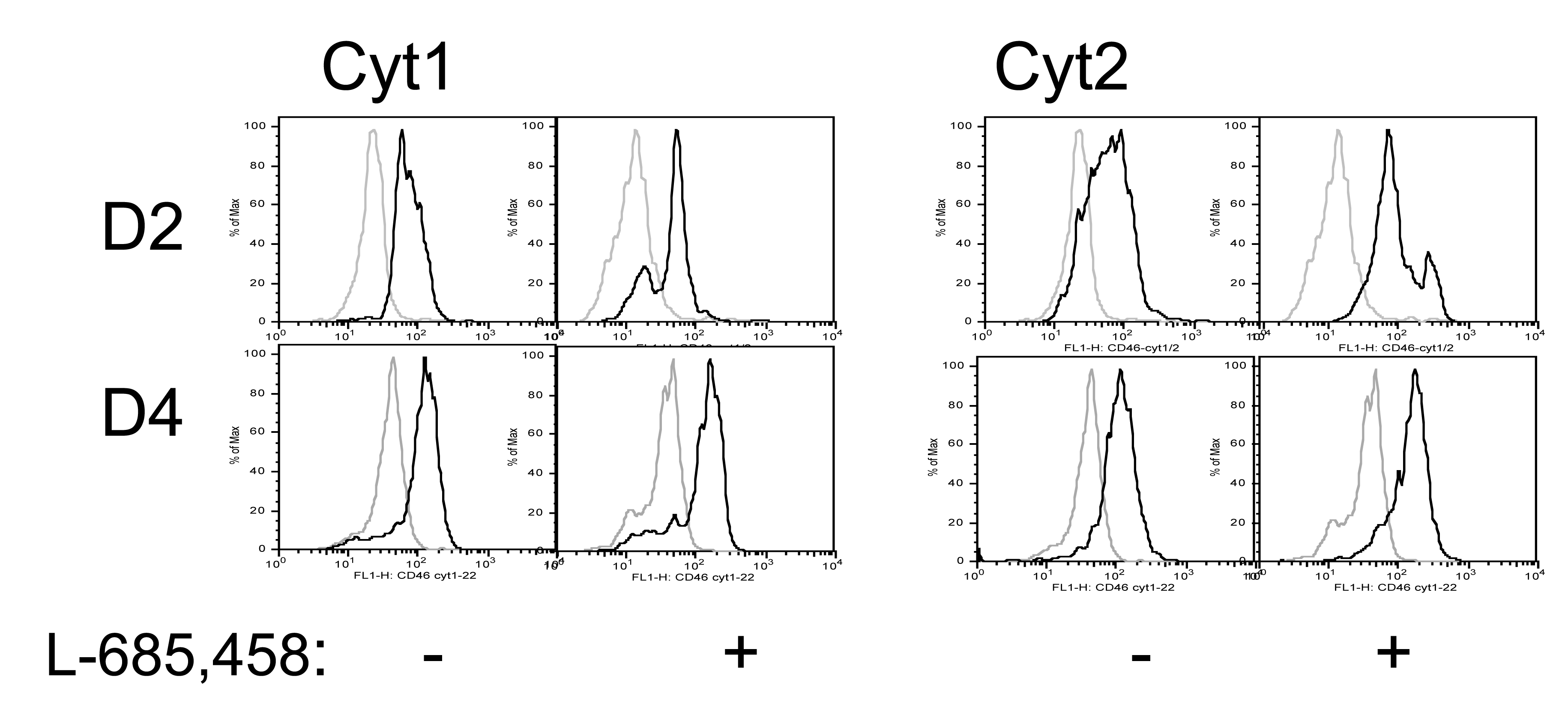

Supplement: Figure S3 — Inhibition of P/γS increases the levels of Cyt1/Cyt2 expression. CD4+ T cells were stimulated by immobilized anti-CD3/CD46 antibodies for 2 or 4 days in presence or absence of L-685,458, a P/γS inhibitor. The expression of Cyt1 and Cyt2 was then analyzed by flow cytometry. Addition of L-685,458 increases the levels of Cyt1 and Cyt2. (TIFF) [file pone.0016287.s003.tif]

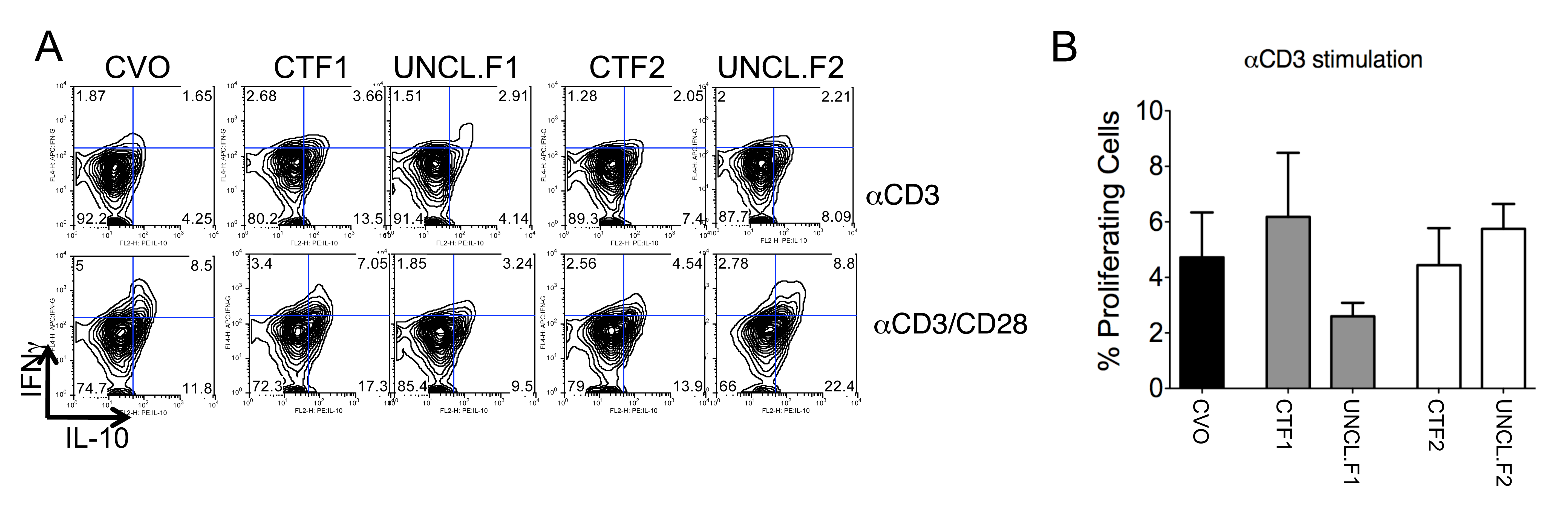

Supplement: Figure S4 — CTF expression can also alter the profile of cytokine produced and the proliferation of CD3-activated transfected T cells. Twenty-four hours post transfection by Amaxa/Lonza with the different CTF constructs, CD4+ T cells were stimulated by immobilized anti-CD3 or anti-CD3/CD28 antibodies. (A) The secretions of IL-10 and IFNγ were assessed by secretion assays (Miltenyi). (B) Proliferation was assessed by flow cytometry (n = 8). In some experiments, anti-CD3 stimulation was very weak – hence we mainly studied the effects of expression of CTF in CD3/CD28 activated T cells. However, in the experiments where it induced T cell activation, we were then able to observe a similar effect of the CTF constructs. (TIFF) [file pone.0016287.s004.tif]

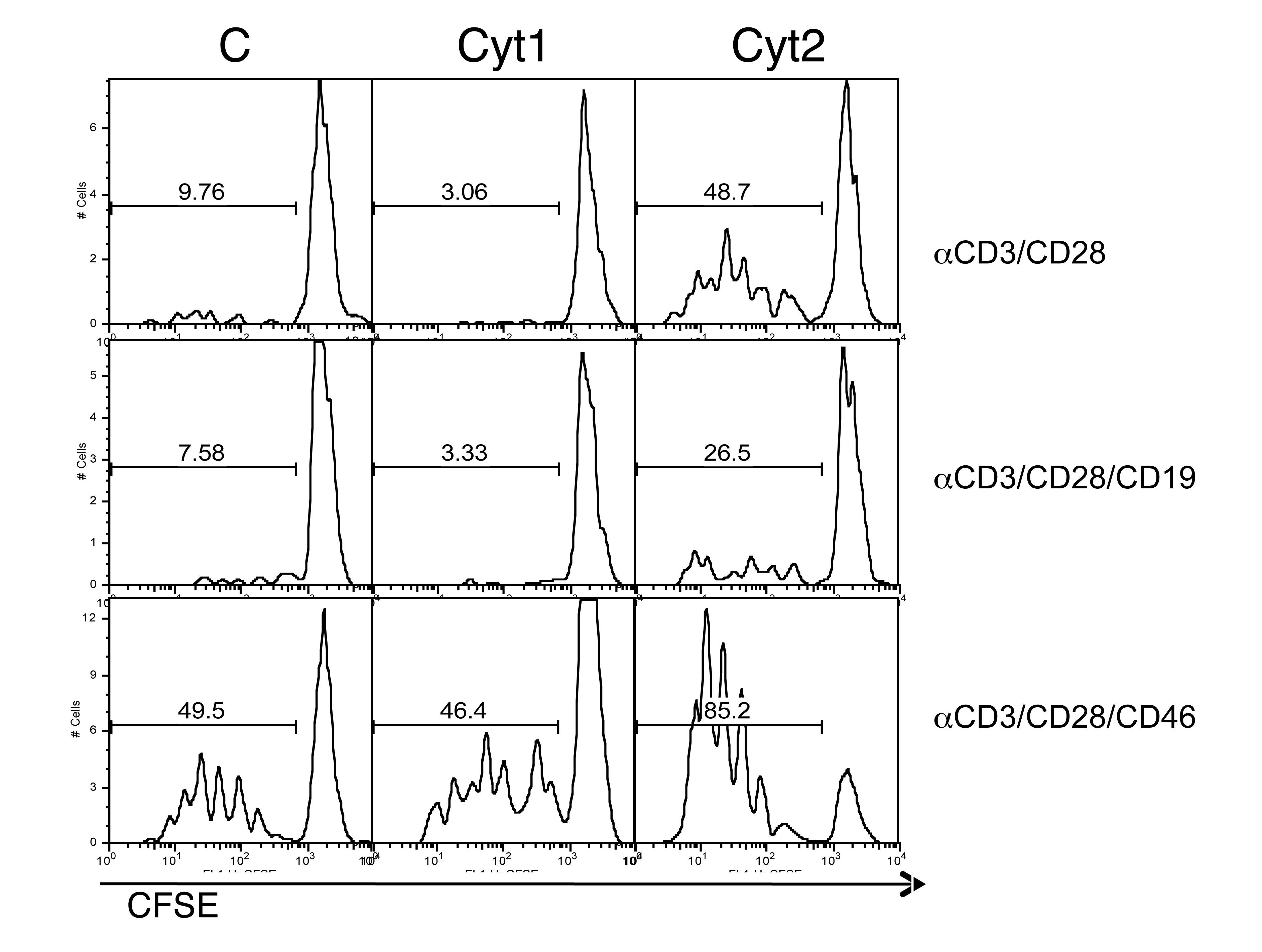

Supplement: Figure S5 — Cyt2 inhibits proliferation of CTLA-4+ cells. Twenty-four hours post transfection by Amaxa/Lonza with the different CD19-CD46 fusion proteins, CD4+ T cells were pre-labeled with CFSE and then stimulated by immobilized anti-CD3/CD28, anti-CD3/CD28/CD19 or anti-CD3/CD28/CD46 antibodies and CTLA-4 expression was determined after 4 days. The proliferation of CTLA-4+ gated cells is shown. (TIFF) [file pone.0016287.s005.tif]

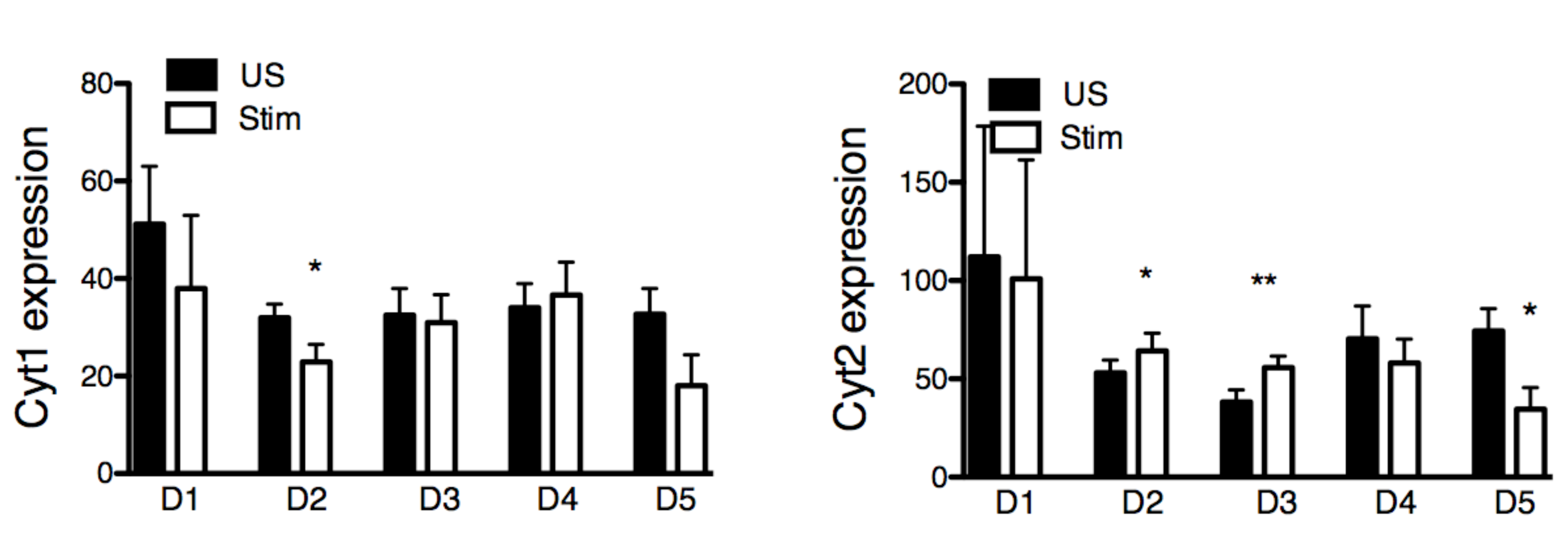

Supplement: Figure S6 — Kinetics of Cyt1 and Cyt2 expression upon CD46-coactivation. Purified CD4+ T cells were left unstimulated (US), or stimulated by immobilized anti-CD3/CD46 (Stim), as indicated, for several days. The expression of the two cytoplasmic tails of CD46 was determined by intracellular staining using specific anti-Cyt1 or Cyt2 monoclonal antibodies (n = 5). (TIFF) [file pone.0016287.s006.tif]
